# Genome Sequence of Fusarium graminearum Strain CML3066, Isolated from a Wheat Spike in Southern Brazil

**DOI:** 10.1128/MRA.00157-20

**Published:** 2020-05-07

**Authors:** Ana K. Machado Wood, Robert King, Martin Urban, Camila P. Nicolli, Emerson M. Del Ponte, Kim E. Hammond-Kosack

**Affiliations:** a Rothamsted Research, Harpenden, Hertfordshire, United Kingdom; b Universidade Federal de Viçosa, Minas Gerais, Brazil; Vanderbilt University

## Abstract

Fusarium graminearum is a global fungal pathogen of wheat and other small grains, causing *Fusarium* head blight (FHB) disease, also known as wheat scab. We report here the annotated genome of a deoxynivalenol/15-acetyl-deoxynivalenol-producing Brazilian strain called CML3066, isolated from FHB-symptomatic wheat spikes collected in 2009.

## ANNOUNCEMENT

The ascomycete fungus Fusarium graminearum is the main pathogen causing *Fusarium* head blight (FHB), an important cereal disease worldwide (1). The F. graminearum genome from a South American strain was not previously available. Here, we report an annotated assembly for strain CML3066 (DON/15-ADON), isolated in 2009 in Rio Grande do Sul state, Brazil (latitude, −28.327, longitude, −51.271). This strain was isolated from a symptomatic wheat spike with 21.5% FHB incidence (2).

Genomic DNA of F. graminearum CML3066 was extracted from mycelia grown for 3 days in potato dextrose broth (PDB) medium using a cetyltrimethylammonium bromide (CTAB) protocol (3) and quantified using a Qubit 2.0 fluorometer (Life Technologies). Libraries were prepared with TruSeq DNA high-throughput (HT) (Illumina) and SMRTbell (PacBio) kits. Genome sequencing was done both on an Illumina HiSeq 2000 platform, producing 100-bp paired-end reads (91× coverage) with no quality control required, and on a PacBio RS II platform with a postquality filter of minimum polymerase read quality of 0.80 and minimum subread length of 500 bp, resulting in 160× coverage with a subread total of 6,392,721,477 bp, 1,358,615 reads, and an *N*_50_ value of 5,621 bp. Default parameters were used for all software unless otherwise noted. The *de novo* assembly was carried out using SOAPdenovo2 v2.0.4 using the Illumina data with a range of k-mer values (61 to 99) and the SMRT analysis portal using the PacBio data. The PacBio assembly was manually gap filled and further scaffolded using Lastz v1.04.03 alignments with the complementary Illumina assembly, resulting in four complete chromosomes from telomere to telomere with no gaps or N bases. Reference sequence statistics were extracted from Geneious v8.1. The genome annotation of CML3066 was done using the MAKER v2.30 (4) annotation pipeline with RepeatMasker v4.50 (5). Gene calls were generated using both AUGUSTUS v2.7 (6) using the F. graminearum species model and GeneMark (7), which was trained using strain PH-1 (8, 9).

The CML3066 assembly is 36,908,675 bp long with a GC content of 47.9%. The CML3066 genome is predicted to contain 14,188 genes, 286 of which are not present in the PH-1 genome. Using the PH-1 reference, a minimum of 80% of the length of the gene was required to have mapped reads to be considered present. Single nucleotide polymorphism (SNP) calling was performed with SAMtools using default settings. SNP effects were predicted using SnpEff 4.2. Comparison of SNP frequencies along all four chromosomes of both CML3066 and PH-1 (8, 10) revealed that all telomere proximal regions displayed the highest SNP density windows. In addition, chromosomes 1, 2, and 4 were found to have one or two large interstitial regions with a high SNP density. To predict secreted proteins, Blast2GO v3.2 was used to identify signal peptide and transmembrane domains. Prediction of glycosylphosphatidylinositol (GPI)-anchored membrane proteins, cellular protein localization, and effectors was performed using Big-Pi, WoLF PSort and ProtComp v9.0 (Softberry), and EffectorP v1.0 (11–14), respectively. The secretome was predicted to contain 874 genes. A genome comparison between CML3066 and the reference strain PH-1 is summarized in Table 1.

**TABLE 1:** Genome sequence assemblies for F. graminearum strains PH-1 and CML3066

Characteristic	Value for strain:	
	PH-1^*a*^	CML3066
Genome size (bp)^*b*^	36,663,736	36,908,675
No. of chromosomes	4	4
GC content (%)^*c*^	48.2	47.9
No. of spanned gaps	0	0
No. of predicted genes	14,145	14,188

a Reannotated genome (10).

b Including all scaffolds and the mitochondrial genome but excluding the large repetitive sequence at the carboxyl end of chromosome 4.

c Excluding the mitochondrial genome.

### Data availability.

The raw data and assembled/annotated sequences have been deposited in the European Nucleotide Archive (ENA). The study accession number is PRJEB12819. The accession numbers for the assembled chromosomes and mitochondrial genome are LT222053 to LT222057. The secretome and effector predictions can be found at https://github.com/ana321wood/Secretome_CML3066_Feb2020/blob/master/Secretome_CML3066_Feb2020_AMW.txt.
